# New Frontiers on ER Stress Modulation: Are TRP Channels the Leading Actors?

**DOI:** 10.3390/ijms24010185

**Published:** 2022-12-22

**Authors:** Vincenzo Vestuto, Veronica Di Sarno, Simona Musella, Giorgio Di Dona, Ornella Moltedo, Isabel Maria Gomez-Monterrey, Alessia Bertamino, Carmine Ostacolo, Pietro Campiglia, Tania Ciaglia

**Affiliations:** 1 Department of Pharmacy, University of Salerno, Via G. Paolo II, 84084 Fisciano, SA, Italy; 2 Pineta Grande Hospital, Via Domiziana, km 30/00, 81030 Castel Volturno, CE, Italy; 3 Department of Pharmacy, University of Naples Federico II, Via D. Montesano 49, 80131 Napoli, NA, Italy; 4 European Biomedical Research Institute of Salerno, Via S. De Renzi 50, 84125 Salerno, SA, Italy

**Keywords:** ER stress, UPR, TRP channels, cancer, inflammation, neurodegeneration, metabolic diseases

## Abstract

The endoplasmic reticulum (ER) is a dynamic structure, playing multiple roles including calcium storage, protein synthesis and lipid metabolism. During cellular stress, variations in ER homeostasis and its functioning occur. This condition is referred as ER stress and generates a cascade of signaling events termed unfolded protein response (UPR), activated as adaptative response to mitigate the ER stress condition. In this regard, calcium levels play a pivotal role in ER homeostasis and therefore in cell fate regulation since calcium signaling is implicated in a plethora of physiological processes, but also in disease conditions such as neurodegeneration, cancer and metabolic disorders. A large body of emerging evidence highlighted the functional role of TRP channels and their ability to promote cell survival or death depending on endoplasmic reticulum stress resolution, making them an attractive target. Thus, in this review we focused on the TRP channels’ correlation to UPR-mediated ER stress in disease pathogenesis, providing an overview of their implication in the activation of this cellular response.

## 1. Introduction

TRP channels represent key sensors for homeostasis maintenance in cellular environments, considering their capability to finely regulate ion balance and to modulate effectors activity. Dysregulation in TRP activity or expression is involved in the genesis of several pathological conditions ranging from neuropathic pain [1], neurodegenerative diseases [2], overactive bladder syndrome [3] to cancer [4]. Several efforts in the research field were made to understand the correlation between TRP channels aberrant activity and disease onset, while poor knowledge of their implication in endoplasmic reticulum (ER) stress development are reported. Given the increasing number of studies about the cellular mechanisms generating these phenomena, we considered it interesting to summarize the latest discoveries in this area.

### 1.1. ER Stress and UPR Overview

The ER represents one of the most functional and extended cell organelles responsible for multiple biological functions. Calcium storage, biomolecules synthesis, protein folding, post-translation modifications and transport, are some of the roles played by ER, contributing to cellular homeostasis. Moreover, ER provides critical features in intra- and intercellular signaling events, through its tubular network, harboring numerous membrane contact sites with plasma membrane, mitochondria, endosomes, Golgi apparatus, peroxisomes and lipid droplets [5,6].

Although all biological processes are finely regulated, there is a wide number of factors, such as nutrient deprivation, DNA damage, calcium depletion, oxidative stress, hypoxia, pH variations, etc., which can cause perturbance in the ER environment, resulting as the so-called ER stress that is characterized by misfolded and/or unfolded proteins accrual. The ER stress often represents a severe issue, since continuous and unresolved ER stress events can lead to cell death in response to the production of pro-apoptotic factors [7]. Nonetheless, when this state is well controlled by the cellular compensatory machinery and the perturbations are overcome, cells’ condition improves, enabling survival. The adaptive signaling response to ER stress is known as unfolded protein response (UPR) and is a self-protective mechanism acting through different routes: (1) decrease in protein translation, (2) transcription of genes encoding factors involved in ER protein folding and clearance (e.g., HRD1, BiP, SEL1L, Herp) and (3) the ER-associated protein degradation (ERAD) process by which misfolded and unfolded proteins are brought back to the cytosol, where they will subjected to the ubiquitin-proteasome system [8,9].

UPR can be decisive to determine cell fate: such as a two-faced Janus, on one side it rescues the cell from a mild or short-term stress, by restoring the ER proteostasis, whilst on the other side, if ER stress is prolonged and overwhelms the UPR, the pro-survival activity turns into a pro-apoptotic response [10,11]. More insights are required for the full understanding of the mechanisms regulating the UPR pathway. However, several studies have clearly described the link between ER stress and multiple physio-pathological states, and, particularly, the involvement of the UPR in cell stress signals and degradation pathways such as autophagy and in the ubiquitination-proteasome system [12,13].

**Figure 1:**
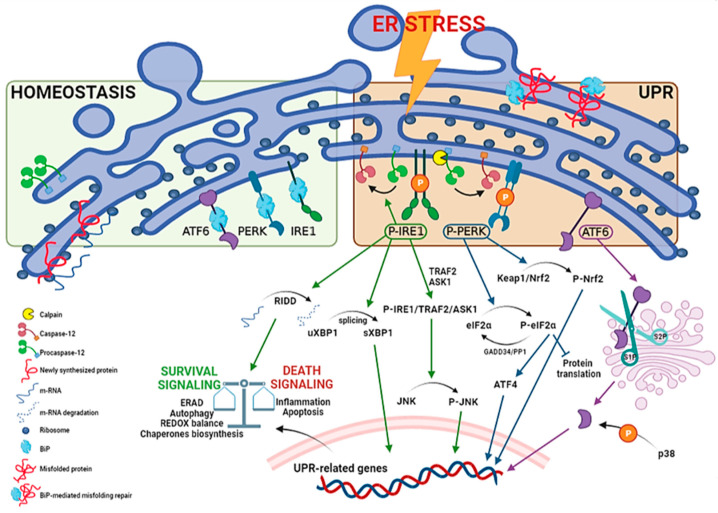
Unfolded protein response pathways. ER stress may be generated by different phenomena that trigger the UPR. The process requires activation by dimerization and autophosphorylation for PERK and IRE1, and intermembrane proteolysis for ATF6 (90 KDa to cleaved form, 50 KDa). Furthermore, UPR activates ER-resident caspase-12. The precise mechanism is not clear, but various processes including interaction with calpain, a calcium-activated cysteine proteases, or activation of the P-IRE1 pathway may contribute to its activation. P-IRE1 induces pro-survival genes to increase protein folding and ERAD. This is allowed through regulated IRE1-dependent decay (RIDD), which degrades several mRNAs, reducing the ER load, as well as IRE1-dependent splicing of uXBP1 to the active spliced factor sXBP1. If ER stress persists, pro-apoptotic genes are activated by a complex of IRE1 with TRAF2 and ASK1, leading to JNK phosphorylation (JNK activation can be distinguished in an early and transient antiapoptotic and a later phase, that coincides with activation of caspases). P-PERK activates, in the short-term, a pro-survival response by eIF2α phosphorylation, leading to the inhibition of protein translation. Furthermore, P-PERK triggers the Nrf2-based antioxidant pathway due to Keap1/Nrf2 complex phosphorylation. Simultaneously, ATF4 transcription leads to pro-survival genes (e.g., ERAD, autophagy pathways). In the long-term, there is the activation of pro-apoptotic genes, such as CHOP, and protein translation reactivation by GADD34/PP1 complex that dephosphorylates P-eIF2α. ATF6 is transported to the cis-Golgi for cleavage by site 1 and site 2 proteases (S1P, S2P), resulting in an active cleaved form (cATF6) that acts as transcription factor to enhance folding and ERAD genes. p38 MAPK may modulate cATF6 activity by phosphorylation.

UPR regulators consist in a set of three ER membrane-associated proteins, acting as ER stress sensors: protein kinase RNA (PKR)-like endoplasmic reticulum kinase (PERK), inositol-requiring transmembrane kinase/endoribonuclease 1 (IRE1) and activating transcription factor 6 (ATF6) (Figure 1).

These proteins contain domains located into the ER lumen, associated with the cytosolic effector domains and acting as ER stress sensors. Each UPR sensor is inactive due to the binding to the ER luminal chaperone glucose-regulated protein 78 (GRP78), also called binding immunoglobulin protein (BiP). When BiP is removed from the three ER stress transducers, it binds to misfolded and/or unfolded proteins accumulated in the ER to facilitate their folding and assembly, and to repair misfolding and aggregation. At the same time, the separation of BiP from the sensors leads both to proteins and UPR activation [14,15,16].

**Figure 2:**
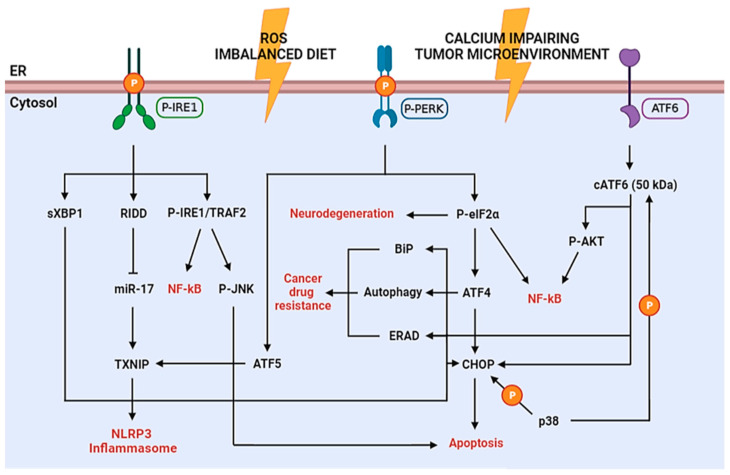
Schematic diagram of the UPR signaling in diseases. P-IRE1 and subsequent sXBP1 directly binds the promoters of UPR genes, which may lead to cell death (CHOP) or survival (ERAD, autophagy, BiP) depending on the intensity of the ER stress. IRE1 activates RIDD-dependent degradation of miR-17, which in normal conditions represses TXNIP, thus enabling increased TXNIP levels and NLRP3 inflammasome activation with consequent inflammatory cytokines production. Furthermore, TXNIP can be induced through the P-PERK–ATF5 pathway to induce inflammasome activation. Moreover, P-IRE1 phosphorylates TRAF2 leading to NF-kB activation (inflammation pathway) or JNK activation (apoptosis pathway). P-PERK phosphorylates eIF2α stopping the protein synthesis. This causes suppression of the synthesis of essential synaptic proteins, leading to neurodegeneration, and inflammation pathway through NF-kB activation. On the other hand, ATF4 expression enhances; this transcription factor represents a double-edged sword since it may activate apoptotic genes (e.g., CHOP, GADD34, caspases), but also pro-survival genes (e.g., BiP and other chaperones, SEL1L, autophagy genes) causing cancer drug resistance in tumor cells. The cleaved form of ATF6 (cATF6) may activate pro-survival or apoptotic response and inflammation pathway (NF-kB), similarly to ATF4. Furthermore, p38 can also phosphorylate CHOP and cATF6 to enhance their activity.

PERK is activated by autophosphorylation and dimerization upon ER stress. It enables a short-term response with the purpose of a pro-survival outcome by inhibiting protein translation, through phosphorylation of the α subunit of the eukaryotic translation initiation factor (eIF2α). The resulting P-eIF2α, in turn tentatively arrests most protein synthesis due to its association with the guanine nucleotide exchange factor eIF2B, by blocking its activity and, thus, causing an arrest in transient protein translation. On the other hand, the synthesis of the activating transcription factor 4 (ATF4) is increased. ATF4 is crucial for pro-survival genes activation and is aimed to maintain redox balance, given that PERK-dependent phosphorylation triggers dissociation of Nrf2/Keap1 complex. The nuclear factor erythroid 2-related factor 2 (Nrf2) represents a potent transcription factor involved in the antioxidant response. Under basal conditions, it is inactivated by the Kelch-like ECH associated protein 1 (Keap1) by degradation through the cullin3/ring box 1-depedent ubiquitin ligase complex. During ER stress, P-PERK phosphorylates Keap1 inducing conformational changes that prevent the binding of de novo produced Nrf2. Therefore, newly translated Nrf2 can migrate into the nucleus to activate antioxidant gene transcription promoting survival [17,18,19]. In the long-term, if ER stress persists and is not resolved, pro-apoptotic factors, such as C/EBP homologous protein (CHOP), are activated by ATF4, inducing the growth arrest and DNA damage-inducible protein 34 kDa (GADD34). This protein forms a complex with protein phosphatase 1 (PP1) leading to the dephosphorylation of P-eIF2α, and thus a suicidal response via the cellular protein synthesis reactivation [20,21,22] (Figure 1). Furthermore, p38 MAPK mediates phosphorylation of two adjacent serine residues (78 and 81) of CHOP, enhances its ability to function as a transcriptional activator, inducing apoptosis or different inhibitory effects [23] (Figure 2).

IRE1 is also activated by autophosphorylation and oligomerization and induces pro-survival genes to enhance ERAD and protein folding. This mechanism is allowed through IRE1 endoribonuclease activity that converts uXBP1 (unspliced X-box binding protein 1) mRNA to the transcription factor sXBP1 (spliced form) [24]. Upon prolonged ER stress, the continuous IRE1-induced RNase activity becomes less specific, resulting in a process of multiple mRNAs degradation called RIDD (regulated-IRE1 dependent decay). If ER stress is not rescued, P-IRE1 generates a complex with TNF receptor-associated factor 2 (TRAF2) and apoptotic-signaling kinase-1 (ASK1) to phosphorylate JNK (c-Jun N-terminal kinase)—in this way, apoptosis is triggered [25,26,27] (Figure 1). IRE1 signaling may also promote cell death by activating CHOP (sXBP1 pathway) (Figure 2) or caspase-12 (Figure 1), an ER-resident and UPR-activated caspase, since ER stress disrupts the interactions between TRAF2 and procaspase-12, probably due to P-IRE1 binding to TRAF2, which in turn leads to the conversion of procaspase-12 into the active form [28,29].

Finally, ATF6, upon release from BiP, traffics to the Golgi compartment, where it is cleaved to an active transcription factor (cATF6) by serine protease site-1 (S1P) and metalloprotease site-2 (S2P). The activated ATF6 induces the upregulation of ER stress-responsive genes, including BiP and CHOP, protein folding and ERAD genes. It is also well known that active ATF6 and its transcriptional activity are modulated by p38-dependent phosphorylation [30,31,32] (Figure 1).

### 1.2. ER Stress in Diseases Pathogenesis

UPR is activated in several disorders, including inflammation, diabetes, metabolic and neurodegenerative diseases, as well as in cancer [33,34,35,36,37,38,39]. UPR can have contrasting effects being either cell protective or cell destructive depending on the strength or duration of the insult [40,41].

It is well known that the UPR pathways are chronically activated in cancer since tumor cells are challenged by microenvironments. Acidosis, hypoxia and hypoglycemia, for instance, lead to the induction of protective module of ER stress. This is supported by the presence of permanently elevated levels of pro-survival GRP78 protein in many tumor cells (Figure 2), exerting protective effects against the cytotoxicity of several chemotherapeutics, such as taxanes, anthracyclines and imidazotetrazines [42,43,44]. On the contrary, CHOP, showing opposite functions than GRP78, generally is not significantly expressed in tumors, despite low and chronic ER stress levels, because the pro-survival action prevails and GRP78 inactivates the ER transmembrane signaling components PERK, IRE1 and ATF6 [45].

Progression of type 2 diabetes requires an increase in pancreatic β-cells for insulin production in order to attenuate insulin resistance. This causes an augmented processing of proinsulin to insulin in the ER that, combined with an enhancement of free fatty acids and glucose, triggers chronic ER stress. If these conditions are maintained for long-term periods, as for obese patients and people with unbalanced diets, chronic ER stress may lead to β-cell death, thus initiating a vicious circle of exacerbated hyperglycemia. Instead, high glucose levels, compatible with diabetes and obesity, induce PERK and eIF2α phosphorylation, concomitant inhibition of protein synthesis, activation of JNK and ATF6 and overexpression of ATF4 and CHOP in β-cells (Figure 2). Moreover, recent evidence suggests that free fatty acids, in particular palmitate, activate the ER stress response in vitro [46,47].

During inflammatory response, ROS production notably increases, impairing ER redox balance and disulfide bond formation. It has been shown that extracellular sources of ROS can induce ER stress, through depletion of Ca^2+^ in the ER. Moreover, ROS can also promote NLRP3 (NLR family, pyrin domain containing 3) inflammasome activation via RIDD (IRE1 pathway) and/or transcription of the activating transcription factor 5 (ATF5) (PERK-eIF2α pathway) via thioredoxin-interacting protein (TXNIP) activation. TXNIP is normally untranslated due to miR-17, which is degraded by RIDD under ER stress conditions. Additionally, ROS trigger inflammatory responses by enhancing the phosphorylation of IκB to induce nuclear factor-κB (NF-κB) signaling by the interaction of IRE1 with TRAF2, phosphorylation of AKT by cleaved ATF6 or translation attenuation by PERK-dependent phosphorylation of eIF2α. The process leads to a decreased expression of both IκB and NF-κB. However, given the shorter half-life of IκB, the resulting increase in NF-κB/IκB ratio prompts inflammation [48,49,50,51] (Figure 2).

UPR signaling also plays a role in neuronal physio-pathological processes and can influence higher brain functions. Accordingly, the accumulation of misfolded proteins is a typical occurrence in many neurodegenerative disorders [52].

Degeneration of substantia nigra dopaminergic neurons and the presence of α-synuclein containing Lewy bodies are peculiar to Parkinson’s disease (PD). α-synuclein is expressed in synaptic vesicles and nervous cell membranes; alterations in α-synuclein post-translational modifications are associated to missense mutations causing dominant familial PD. Thus, these aberrations can cause protein misfolding due to the shift from the predominantly α-helix to β-sheet conformation, followed by plaque formation [53]. For instance, the A53T mutation is related to UPR activation as shown by increased expression of BiP, CHOP and P-eIF2α. Inhibition of eIF2α phosphorylation rescues the A53T cells from apoptosis, suggesting that the activated UPR may promote cell death in these disorders [54] (Figure 2). Further in vitro evidence displays pro-survival effect of cocoa procyanidins on PD model induced by 6-hydroxydopamine inhibiting UPR via PERK phosphorylation block [36].

Alzheimer’s disease (AD) is an age-associated dementia disorder characterized by the accumulation of extracellular β-amyloid (Aβ) peptides and hyperphosphorylated tau (pTau) protein, leading to intracellular protein aggregation in senile plaques. Ca^2+^ homeostasis is essential to regulate functions of ER chaperones and protein folding. Alterations in Ca^2+^ balance lead to reduced chaperone activity, protein misfolding and initiation of the UPR. Aβ peptides have been shown to cause severe depletion of ER Ca^2+^ storage by triggering release into the cytosol [55,56] (Figure 2).

Amyotrophic lateral sclerosis (ALS) represents a progressive neurological disease that primarily affects motoneurons. Bunina bodies, deposition of ubiquitylated proteins and aggregates of mutant superoxide dismutase 1 (mSOD1) and protein disulfide isomerase (PDI) are prodromic of the disease. Contrasting data suggest that UPR activation may cause ER stress-induced apoptosis or may be neuroprotective by strongly triggering autophagy [57,58] (Figure 2). Therefore, it is evident that cellular mechanisms affecting the balance between the protective and apoptotic response of the UPR are critical in these pathologies.

## 2. TRP and ER Stress

ER-cytosol Ca^2+^ flux exchange is an intricate network of processes mediated by sever-al proteins, mainly including the energy-consuming ATPase SERCA2b [59] together with the transient receptor potential (TRP) channels, involved in the ER calcium influx [60].

Ion channels are crucial in maintaining ion balance inside and outside cells and to transmit signals required for proliferation, migration, invasion, autophagy and adhesion mechanisms. In this regard, calcium, acting as second messenger, can activate or inactivate various regulatory proteins such as chaperones, enzymes and transcriptional factors, in addiction to influencing ER stress and the autophagic process. All these events are highly relevant for the genesis of numerous diseases [61,62,63,64].

The expression and activity of TRP closely control the intracellular Ca^2+^ concentrations and, as a result, all the Ca^2+^-dependent processes. Accordingly, specific TRP channel modulation and localization may influence multiple regulatory pathways, contributing differently to the control of homeostasis and, in turn, determining a specific effect on Ca^2+^ signaling, eventually affecting the endoplasmic reticulum.

For these reasons, we can hypothesize that, under ER stress, TRP channels are regulated as a part of UPR, supervising cell fate: specifically, some TRP channels contribute to ameliorate ER stress (e.g., TRPV6, TRPC1, TRPML1), while others (e.g., TRPV1, TRPC6, TRPC3) increase stress promoting cell death.

The TRP superfamily includes seven major subfamilies with different expression and localization: TRPC (canonical), TRPV (vanilloid), TRPA (ankyrin), TRPM (melastatin), TRPP (polycystin), TRPML (mucolipin) and TRPN (named NOMPC, no mechanoreceptor potential C). TRPs are nonselective cation-permeable channels and behave as the major actors for establishing the calcium balance inside the cell environment. The channels activation is mediated by a variety of stimuli, ranging from temperature, mechanical and osmotic stresses to chemical substances and signaling molecules [65]. TRP’s activation is mediated by G protein-coupled receptors (GPCRs) that activate phospholipase C (PLC), hydrolyzing phosphatidylinositol 4,5-bisphosphate (PIP_2_) to generate soluble inositol 1,4,5-trisphosphate (InsP_3_) and diacylglycerol (DAG), which work as the main effectors of TRPs triggering [66].

Currently, only the crystal structure of TRPV6 mutant has been solved, considering the complexity in acquiring well-diffracting crystals of this protein’s family [67]. Nevertheless, cryo-electron microscopy (cryo-EM) furnished several details about TRP channel structural biology revealing the key structural determinants for their activity and pharmacology. In particular, the structures for TRPV1, TRPA1, TRPV2, TRPP2, TRPN, TRPML3, TRPML1, TRPM8, TRPM4, TRPV6 and TRPV5, associated to ligands or not, are deposited (http://www.emdatabank.org, accessed on 1 November 2022).

The TRP channel transmembrane domain (TMD) shows remarkable similarities with voltage-gated ion channels, as the Kv channels, with four subunits organized in homo- or hetero-tetramers arranged around a central ion permeation pore. Each subunit is composed of six transmembrane α-helices (S1–S6) with S1–S4 forming the voltage-sensing-like domain (VSLD) and S5–S6 and pore helixes (PH) forming the pore domain. The NH_2_ and COOH-terminal residues are located in the intracellular side. The VSLD subunits adopt a “domain-swap” organization, where each VSLD interacts with the PD from the flanking subunit. Despite the highly conserved architecture of the TMD, each TRP channel subfamily exhibits very low sequence homology, reflecting their diverse physiological capability to respond to exogenous and endogenous stimuli [65].

Recently, evidence suggest the connection among the outbreak of several diseases, such as ischemia, diabetes, cancer and neurodegenerative pathologies, with ER stress and, finally, with TRP activity alterations or aberrant expression. Among TRP channels TRPV, TRPC and TRPM, subtypes play a major role in the cellular mechanisms leading to ER stress development.

### 2.1. TRPV in ER Stress

TRPV receptors consist of six members organized into four groups sharing common structure and functional role: TRPV1/TRPV2, TRPV3, TRPV4 and TRPV5/6 [68].

One of the main characterized members of the TRPV subfamily is TRPV1, tangled in detection of nociceptive stimuli and responsive to chemicals such as capsaicin and inflammatory mediators (leukotriene B4, LTB4), temperature or pH modifications [69]. The transient receptor potential vanilloid 1 (TRPV1) works as a nonselective ligand-gated cation channel with high calcium permeability, showing an extensive localization in sensory neurons but also in many other different cell types [70]. Furthermore, confocal imaging revealed high TRPV1 subtype concentration in ER [71]. Recent studies suggest that TRPV1 agonists and antagonists display anticancer activities in diverse types of human cancer cells, including colorectal and oral cancer [72,73] and could be taken into account for the development of more effective therapies. However, on the basis of current studies, TRPV1’s role in ER stress is controversial.

**Figure 3:**
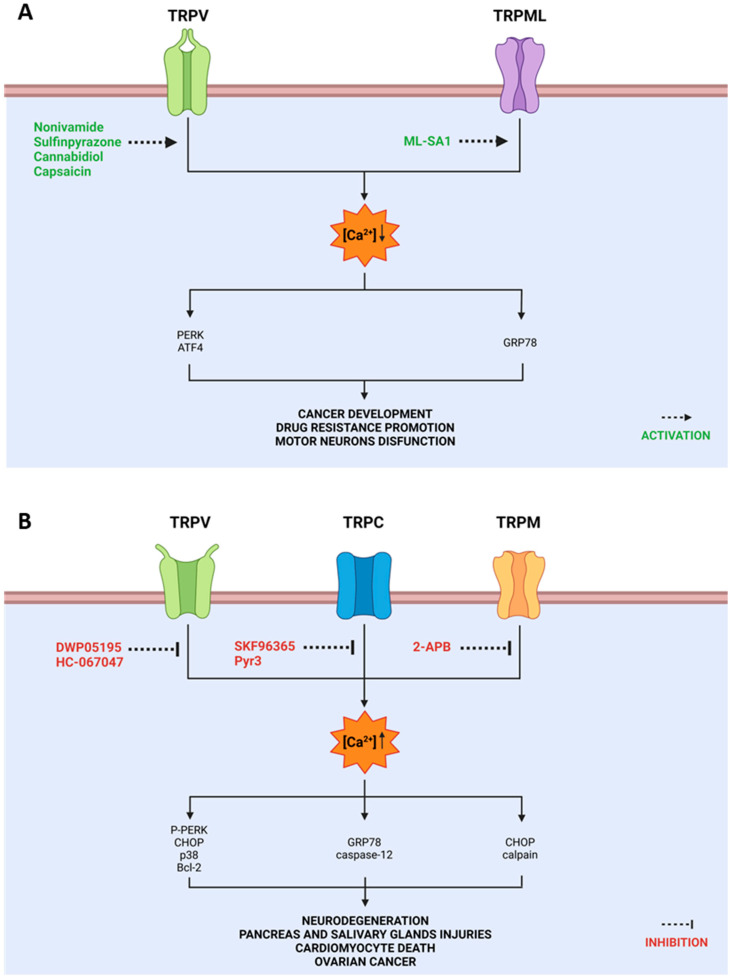
Activation (**A**) and inhibition (**B**) of TRP channels involved in ER stress modulation. The opening or blocking of these channels affects the flow of intracellular calcium by modulating various biological functions including drug resistance, cancer survival, neurodegeneration and tissue injuries. Color code: antagonists and inhibitors of TRP expression are indicated in red; agonists are reported in green.

TRPV1 agonists, such as nonivamide and sulfinpyrazone, interfere with ER calcium homeostasis (Figure 3) through a complex mechanism involving the expression of ATF4 that promotes downstream PERK-dependent signaling pathway causing ER stress responses and cytotoxicity [74]. Indeed, in TRPV1-overexpressing cells treated with nonivamide, a significant increase in cytosolic calcium, due to its release from ER storages, was observed along with reduced cell viability [71].

Cannabidiol, a TRPV1 activator, is reported to improve Ca^2+^ influx and to increase ROS production and cell death in the breast cancer cell line. On the other hand, concomitant treatment with a TRPV1 antagonist is reported to increase cell viability [75].

DWP05195, a novel TRPV1 antagonist (Figure 3), increased the accumulation of intracellular ROS, leading to ER stress through p38 activation. Upon ER stress, the expression of CHOP was upregulated, inducing apoptosis through both intrinsic and extrinsic pathways in human ovarian cancer cells [76].

Moreover, the treatment of nasopharyngeal carcinoma cells with capsaicin, an active component of chili peppers, leads to ROS production and gives rise to ER stress (Figure 3). These events are followed by an improvement in intracellular [Ca^2+^] and mitochondrial membrane depolarization, also resulting in the release of cytochrome c and activation of caspase-9 and -3. In addition, IRE1, CHOP, GRP78 expression and caspase-12 activation [77] have been found after capsaicin administration [78].

TRPV4 is a weakly selective Ca^2+^ permeable channel, working as an osmolality-sensitive ion channel, but also as a thermoreceptor [79]. It is widely distributed in several cell types, including primary afferent epithelial cells [80], keratinocytes [81], dorsal root ganglion neurons, hippocampal neurons and urothelial cells [82,83].

Considering the TRPV4 channel’s pivotal role in neuronal cells’ calcium-levels control, their disruption is strictly connected to neurodegenerative disorders such as Parkinson disease. The TRPV4 channel results overexpressed in rat pheochromocytoma tumor cell line mediating oxidative stress and mitochondrial dysfunction, leading to ER stress-induced apoptosis [84].

Shen et al. reported that TRPV4 is overexpressed in a mouse model of intracerebral hemorrhage, causing disruption of Ca^2+^ homeostasis and UPR activation. The administration of HC-067047, a selective TRPV4 antagonist (Figure 3), decreases UPR and modulates the PERK/CHOP/Bcl-2 signaling pathway, leading to the increase in neuron survival rate [85].

Given the detected expression in the hippocampus, TRPV4 correlation to ER stress was analyzed in neurological complications involving this region, characterized by hyper neuroinflammation, such as sepsis-associated encephalopathy. Zhong et al. demonstrated that TRPV4 inhibitor HC-067047 prevents the lipopolysaccharides-induced neuronal damage exerting anti-ER stress effects, as showed by the decreased expression of ER stress markers, such as P-IRE1, P-eIFα, P-PERK, BiP and CHOP [86].

TRPV6 is widely expressed in all the tissues involved in transcellular Ca^2+^ transport, including colon, duodenum, kidney and in glandular exocrine tissues [87,88], and its overexpression has been linked to several epithelial cell cancers, including prostate, breast, ovary, colon and pancreatic cancers [89,90,91].

TRPV6 was localized downstream of the UPR signaling cascade in human embryonic stem-cell-derived cardiomyocytes, where it seems to play a protective role against ER stress-mediated apoptotic death. The underlying cytoprotective pathway involves ATF6 acting as TRPV6 activator, which subsequently stimulates JNK and reduces ER stress events and cell death [92].

### 2.2. TRPC in ER Stress

Transient receptor potential canonical (TRPC) channels include seven members grouped into four subfamilies (TRPC1, TRPC2, TRPC4/5 and TRPC3/6/7) on the basis of the sequence homology and functional similarities. TRPC channels are unselective cation permeable channels and their structure resembles the TRP superfamily architecture with six helices forming the tetramer subunits surrounding the pore permeable domain. Channel activation is mediated by multifactor events, such as depletion of intracellular endoplasmic reticulum Ca^2+^ storages [93], diacylglycerol produced upon phospholipase C activation [94] and Gq protein-coupled receptor stimulation [95].

TRPC channels are mainly expressed in the cell plasma membrane of different tissues, including brain areas, muscle tissues, skeletal muscle, aorta, heart and diaphragm [96].

TRPC channels’ activation mainly leads to Na^+^ and Ca^2+^ entry, causing membrane depolarization and cytosolic [Ca^2+^] increase, with relevant effects on cellular functions [97]. In fact, genetic mutations of TRPC channels are correlated to the genesis of neurodegenerative pathologies, as familial focal segmental glomerulosclerosis (FSGS) [98]. Diverse TRPC isoform activity is also correlated to ER stress, as reported below.

TRPC1 channel’s key role in these biological events was evidenced by the relationship between its expression and ER stress development: endogenous store-operated Ca^2+^ entry activity, critical for maintaining ER Ca^2+^ levels, is strictly dependent on TRPC1. Indeed, a decreased TRPC1 activity leads to ER stress and cell death, while TRPC1 overexpression is associated with the reduction of ER Ca^2+^ levels and unfolded protein response in dopaminergic neurons [99]. Recent evidence also suggested the TRPC1 protective role in salivary gland cells: TRPC1 silencing induces overexpression of GRP94 and CHOP, and apoptotic markers, mainly caspase-3 and Bax [100]. TRPC1’s protective function was also demonstrated in murine macrophage cell line Raw 264.7, where treatment with the antagonist SKF-96365 (Figure 3) blocked Ca^2+^ entry via the ORAI1/TRPC1/STIM1 complex, promoting cytokine production, autophagy and caspase activation, and, finally, inducing apoptosis [101]. In adipocytes, endogenous Ca^2+^ entry is dependent on TRPC1, and reduction of its activity inhibits adipocyte differentiation, as well as adipokine secretion, leading to metabolic dysfunctions and obesity-associated syndromes [102].

In a mice model of acute pancreatitis, inhibition of TRPC3 by the selective antagonist Pyr3 [103] decreased calcium influx by about 50%, protecting pancreas and salivary glands from Ca^2+^-dependent injury [104].

In podocytes, TRPC6 channels mediate calcium entry, resulting in activation of endoplasmic reticulum stress and apoptosis mechanisms upon albumin overload. In cells transfected with TRPC6 siRNA, the expression of GRP78, caspase-12 activation and podocyte apoptosis were abolished [105].

Moreover, TRPC3 and TRPC6 expression induced by doxorubicin (DOX) treatment causes Ca^2+^ overload and ER stress in cardiomyocytes. On the other hand, treatment with the TRPC blocker SKF-96365 significantly inhibits DOX-induced intracellular Ca^2+^ overload and decreases ER stress-related protein expression [106].

### 2.3. TRPM in ER Stress

TRPM channels include eight members grouped into four classes (TRPM1/TRPM3, TRPM2/TRPM8, TRPM4/TRPM5 and TRPM6/TRPM7) sharing structural arrangement but differing for tissue localization and physiological activity [107]. However, within the TRPM subfamily some isoforms show peculiar features, such as TRPM2 containing a nudix hydrolase domain (NUDT9-H), and TRPM6 and TRPM7, presenting serine/threonine kinase domains in their C-termini [108]. Several studies demonstrated that TRPM6 and TRPM7 channels’ function and regulation are clearly dissociated from autophosphorylation or kinase activity [109]. Otherwise, the TRPM2 NUDT9-H domain seems to play an important role in channel activation in the presence of Ca^2+^, given the abolished channel gating when this fragment is deleted [110].

Nowadays, limited evidence was collected about TRPM’s role in ER stress outcome, and, herein, we reported recent findings disclosing their implications in ER stress signaling pathway activation, focusing on the main isoforms involved in ER homeostasis regulation.

The first characterized member of the TRPM subfamily is TRPM1, widely expressed in retina ON-bipolar cells [111] and downregulated in aggressive metastatic melanoma cells, thus representing a potential marker for melanoma clinical diagnosis [112].

Irrespective of the extensive colocalization of TRPM1 throughout the ER network, as evidenced in HEK293 and CHO cells transiently co-transfected with TRPM1 and Emerald-Sec61β, an ER membrane marker [113], no further experiments demonstrated its involvement in ER stress progression.

Transient receptor potential cation channel, subfamily M, member 2 (TRPM2) activation is mediated by the generation of adenosine diphosphate ribose (ADPR), promoted in response to reactive oxygen and nitrogen species (ROS/RNS) [114]. This channel is involved in several physiological mechanisms, such as insulin secretion from pancreatic β-cells [115] and inflammation events [116]. TRPM2 implication in ER stress-induction has been reported in stress-induced pericyte injury. The levels of ER stress signaling proteins CHOP, P-JNK and P-PERK were examined in zinc oxide nanoparticles-exposed (ZnO-NP) pericytes upon treatment with TRPM2-siRNA. The siRNA-mediated reduction of P-JNK and CHOP, as well as the reduced degradation of IRE1 and PERK, evidencing TRPM2 expression and ER stress-autophagy pathway, were closely correlated [117].

In APP/PS1 (human amyloid precursor protein/presenilin 1) Alzheimer’s mouse model, the inactivation of TRPM2 was shown to normalize the unfolded protein response and to decrease endoplasmic reticulum stress markers levels, mitigating synaptic loss and inflammatory phenomena [118].

TRPM7, unlike other TRP members, shows a ubiquitously expression [119], acting preferentially as a Mg^2+^ permeable channel, even if it contributes remarkably to intracellular Ca^2+^ and Zn^2+^ homeostasis [120].

Accumulating evidence indicate that TRPM7 is involved in multiple cellular processes including survival, proliferation, differentiation, growth and migration, along with modulation of ER stress-response activation.

TRPM7 channels modulate the expression of caspase-12 and CHOP [121], and their overexpression/upregulation is associated with hyperglycemia-induced ER stress and resultant neuronal cell damage. In a hyperglycemia mouse model, increased levels of Trpm7, INOS, Grp78 and Chop mRNAs were observed and associated to ER stress and apoptosis cascade activation. Silencing TRPM7 inhibited ER stress-associated protein expression, decreasing cytotoxicity in NS20Y cells [122].

ER stress-mediated apoptosis mechanisms have been strongly correlated to TRPM7 imbalance; as shown by Li et al., TRPM7 expression is upregulated in a rheumatoid arthritis rat model, while 2-aminoethoxydiphenyl borate (2-APB) (Figure 3) treatment induces the increase in CHOP and calpain expression, and decrease in caspase-3 expression. These results suggest that TRPM7 inhibitors induced apoptosis through ER stress [123]. The same effect on survival and proliferation was observed in hepatic stellate cells following TRPM7 blockage [124].

One of the best characterized members of the TRPM family is the TRPM8 channel, which is thought to be a promising target in cancer therapy, given its overexpression in several tumors, including breast cancer [125], osteosarcoma [126], squamous cell cancer [127] and prostate cancer [128]. Selective amino-acid-based TRPM8 antagonists are able to inhibit androgen-dependent prostate cancer cell proliferation, migration and invasiveness [129].

Recently, a new TRPM8 isoform was identified in the ER membranes from human prostate cancer cells and keratinocytes, characterized by the presence of four transmembrane domains instead of six (4-TM-TRPM8) [130]. This isoform has a specific role in the regulation of PCA cell death, as demonstrated by qPCR experiments. Expression of ER stress markers, such as BiP, CHOP, PERK and sXBP1, is induced by 4-TM-TRPM8 truncated isoforms suppression, leading to apoptosis in prostate cancer cells [131].

To date, no further evidence of the ER-stress-induced TRPM8 channel has been reported and additional studies are required.

### 2.4. Other TRP Channels in ER Stress

The first member of the mammalian mucolipin TRP channel subfamily (TRPML1) is mainly localized in the membranes of late endosomes and lysosomes (LELs), with high expression levels in the brain, kidney, spleen, liver and heart [132]. TRPML1 physiological functions concern the fusion or fission of vesicles within the endocytic pathway and lysosomal ion homeostasis modulation [133].

Based on its role in lysosomal function and autophagy, TRPML1 was analyzed with respect to neurological diseases, such as ALS. Tedeschi et al. showed that TRPML1 co-localizes with the endoplasmic reticulum Ca^2+^ sensor STIM1 in differentiated mouse motor-neuron-like hybrid cells (NSC-34) playing a protective role against motor neurons ER dysfunction caused by the cyanobacterial neurotoxin beta-methylamino-L-alanine (L-BMAA). Indeed, treatment with ML-SA1, a TRPML1 agonist (Figure 3A), induced a rapid intracellular Ca^2+^ increase, preventing the upregulation of GRP78 [134], which is associated with tumor cells proliferation and drug-resistance promotion [135].

Recent findings demonstrated that TRPA1 and TRPV3 channels’ activity is directly correlated to the ER stress process in an opposite manner. In detail, in human bronchial epithelial cells, TRPA1 activation induces PERK-driven ER stress initiation indicated by incremented expression of pro-apoptotic biomarkers, such as DDIT3 and ATF3, together with cell cycle arrest and cell death. On the other hand, TRPV3 overexpression confers resistance to ER stress, decreasing the associated cell cycle block [136].

## 3. Conclusions and Perspectives

The TRP channels represent key cellular sensors and transducers of physio-chemical stress, playing pivotal roles in a variety of physio-pathological processes. Recent findings suggest their activity not limited to calcium homeostasis maintenance, but also linked to UPR pathways control. TRP localization and function are differently involved in ER stress mechanisms development, ranging from its activation, when overexpressed or up/downregulated in some districts, to its blocking upon inhibitory events, depending on the channel distribution and physiological activity. TRPV, TRPC and TRPM channels seem to represent the main actors in this scenario, working as ER stress inducers or blockers according to the particular cellular mechanism taken into account. Furthermore, other TRP receptors are involved in ER disfunction and its mediator’s expression, but additional studies are needed to elucidate their exact role.

In this review we have reported the recent findings about TRP receptor/ER stress development correlation, focusing on the different interactions within these complex pathways and providing, at the same time, a wide perspective on the cause–effect relationship. The full understanding of TRP involvement in ER stress development may represent a milestone to further deepen the study of these processes and the underlying cellular mechanisms.

Moreover, considering the number of efforts addressed towards TRP channel modulators’ design, this analysis could pave the way for the development of new therapeutics directed against UPR-associated diseases, such as cancer, neurodegenerative and metabolic disorders.

## Data Availability

Not applicable.
